# Association of urinary dysfunction after lower rectal cancer surgery with renal function: a single-center study

**DOI:** 10.1007/s00384-025-04955-1

**Published:** 2025-07-14

**Authors:** Ryosuke Kikuchi, Kazuhito Sasaki, Yusuke Sato, Aya Niimi, Akira Sakamoto, Hiroaki Nozawa, Koji Murono, Shigenobu Emoto, Yuichiro Yokoyama, Kensuke Kaneko, Haruki Kume, Soichiro Ishihara

**Affiliations:** 1 Department of Surgical Oncology, Faculty of Medicine, The University of Tokyo, Tokyo, Japan; 2 Department of Urology, Faculty of Medicine, The University of Tokyo, Tokyo, Japan

**Keywords:** Lower rectal cancer, Postoperative urinary dysfunction, Uroflowmetry test, Postoperative renal dysfunction, Urinary tract complications

## Abstract

**Purpose:**

Urinary dysfunction (UD) is still a major complication after lower rectal cancer (LRC) surgery. Untreated UD is an independent risk factor for renal dysfunction due to repeated urinary reflux and urinary tract infections. However, the relationship between postoperative UD and renal function following LRC surgery remains unclear. In this study, we investigated the impact of UD on renal function post-surgery.

**Methods:**

We retrospectively evaluated 83 patients with LRC who underwent curative resection at our tertiary referral center between April 2015 and December 2018. UD was diagnosed as a post-void residual urine volume ≥ 50 mL using uroflowmetry tests after discharge. We compared the estimated glomerular filtration rate (eGFR) and the incidence of chronic kidney disease (CKD)—defined as an eGFR < 60 mL/min/1.73 m^2^—at 3 years after LRC surgery between the UD and non-UD groups. Patient selection was based on the criteria that excluded those with a history of urogenital interventions or incomplete postoperative follow-up. Statistical analysis used the Mann–Whitney U test for continuous variables, Fisher’s test for categorical data, and multivariate logistic regression to adjust for potential confounders.

**Results:**

Of the 83 patients, 21 (25%) had UD. Patients with UD were older, underwent more extensive surgery, and had significantly longer operation times than those without UD. Within 3 years post-surgery, the UD group experienced a higher incidence of urinary tract complications and CKD, with a notable decrease in eGFR. Additionally, a history of hypertension and UD were identified as independent risk factors for CKD at 3 years post-surgery.

**Conclusions:**

Patients with UD showed a significant decrease in eGFR and were more likely to progress to CKD at 3 years after LRC surgery. These findings indicated that postoperative UD might adversely affect renal function in patients with LRC.

**Supplementary Information:**

The online version contains supplementary material available at 10.1007/s00384-025-04955-1.

## Introduction

The standard surgical procedure for lower rectal cancer (LRC) is total mesorectal excision with autonomic nerve preservation [1]. Combined with minimally invasive surgery, this technique can preserve urinary function [2, 3]. However, urinary dysfunction (UD) after LRC surgery remains a major complication. A recent meta-analysis study on the frequency of UD after rectal cancer surgery demonstrated that 20% of patients experienced UD more than 1 year postoperatively [4]. Despite precautions, autonomic nerve injury during LRC surgery may result in neurogenic bladder [5, 6], leading to complete or incomplete urinary retention with a high post-voided residual volume (PVR) [7].

Previous studies in urology have shown that untreated benign prostatic hyperplasia (BPH) with high PVR leads to complications, including urinary reflux, urinary tract infections (UTI), and hydronephrosis. These complications increase risks for renal dysfunction and chronic kidney disease (CKD) [8, 9], potentially escalating to cardiovascular issues and sudden death [10]. In rectal cancer, it has been noted that some patients develop long-term UD with a high PVR [4, 7, 11], which might adversely affect renal function similar to BPH effects. In contrast, previous studies established no direct link between UD and renal outcomes [12, 13].

The uroflowmetry test is the most widely used tool by urologists to assess UD [14]. Despite its prevalence in evaluating urinary function [14], a notable gap remains in the literature, as no existing studies have specifically explored the impact of UD, as determined via uroflowmetry tests, on renal function following LRC surgery. At our hospital, uroflowmetry tests, including PVR measurements, are performed for patients post-LRC surgery after discharge. To clarify the relationship between renal function and UD after LRC surgery, we investigated the influence of UD, diagnosed using uroflowmetry tests, on renal function in patients with LRC.

## Materials and methods

### Patients

**Fig. 1:**
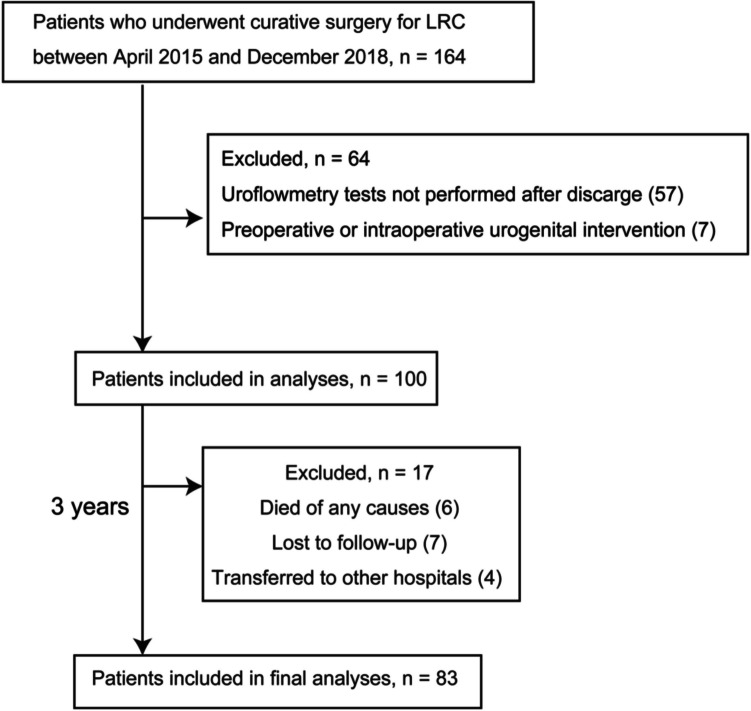
Patient flow diagram. LRC: Lower rectal cancer

This single-center retrospective study was performed using a database collected from our department at the tertiary referral center. Figure 1 shows a flowchart of the study. We initially included 164 patients with LRC, defined as an adenocarcinoma below the peritoneal reflection, who underwent curative resection between April 2015 and December 2018. Two patients had synchronous liver metastases, and one had extra-regional lymph nodes. Of these three patients, two underwent curative resection for metastases at the same time as LRC resection, and one underwent resection before the LRC resection. In accordance with the flow diagram, 81 cases were excluded owing to missing key variables or urogenital intervention, and the remaining 83 cases were analyzed in the present study.

### Procedures and data collection

Tumor location was detected using digital rectal examination, colonoscopy, and barium enema. Computed tomography and pelvic magnetic resonance imaging were used to evaluate the local spread and distant organ metastases of LRC. Patients were staged according to the Union for International Cancer Control, TNM Classification of Malignant Tumours, 8th edition [15]. In addition, we collected other clinical variables, including age, sex, medical history, and blood test results, from medical charts.

Patients with LRC classified as T3 or greater and any N stage underwent neoadjuvant chemoradiotherapy (CRT) [16]. We performed lateral lymph node dissection (LLND) when patients with T3 or greater LRC did not receive CRT, and selectively performed LLND when patients were suspected of having lateral lymph node metastases before CRT [17]. All patients underwent surgery according to the principles of total or tumor-specific mesorectal excision. Surgical procedures included low anterior resection, intersphincteric resection, and abdominoperineal resection, which were performed via open, laparoscopic, or robotic surgery. The creation of a diverting stoma was based on the judgment of the operating surgeons; four patients did not undergo diverting stoma closure. Adjuvant chemotherapy was recommended in accordance with the latest guidelines of the Japanese Society for Cancer of the Colon and Rectum [18]. Chemotherapy regimens were modified at the physician's discretion according to the patient's age, comorbidities, and pathological stage. After LRC surgery, patients were generally followed up with routine blood tests every 3 months and computed tomography every 6 months for up to 5 years [18].

### Evaluation of urinary function and definition of UD

At our hospital, urinary catheters are typically removed on day 5 after LRC surgery, after which PVR is measured in all patients by ultrasonography. If PVR was ≥ 50 mL for two consecutive days, the patient was referred to a urologist.

After discharge, uroflowmetry tests, including PVR measurements by ultrasonography, were performed within 3 months after LRC surgery. Patients were asked to drink fluids and perform the test when they had the urge to void [19]. Urologists at our hospital conducted these evaluations of urinary function. UD was defined as a high PVR (as the presence of ≥ 50 mL on uroflowmetry tests) [9, 20]. Although the voided volume over 150 mL is desirable for uroflowmetry tests [14, 19], several patients could not meet this threshold owing to stoma creation [21]. Patients with UD were allocated to the UD group, and the remaining patients were allocated to the non-UD group. Because patients who required reinsertion of the urinary catheter during hospitalization could not void, uroflowmetry tests were not performed. Physicians referred patients to a urologist after discharge at their discretion if urinary tract complications were suspected.

### Evaluation of renal function and definition of renal dysfunction

Renal function was evaluated by calculating the estimated glomerular filtration rate (eGFR) using the formula recommended by the Japanese Society of Nephrology and Pharmacotherapy [22]:

$$eGFR\;\left(mL/min/1.73m^2\right)=194\;\times\;age-0.287\;\times\;serum\;creatinine\;\left(mg/dL\right)-1.094\;\left(\times0.739\;in\;females\right)$$

CKD was defined as an eGFR of < 60 mL/min/1.73 m^2^ according to the Kidney Disease: Improvement Global Outcomes clinical practice guidelines [12, 23].

eGFR was measured at three time points: before LRC surgery, 1 year post-surgery, and 3 years post-surgery, with patients not undergoing hydration at these assessments. In addition, we selected the time point before LRC surgery as close to the surgery date as possible. We compared the eGFR and incidence of CKD at 3 years after LRC surgery between the UD and non-UD groups [12]. Moreover, to minimize the influence of age and sex on eGFR, we compared the eGFR ratio (ratio of eGFR at 3 years after LRC surgery to that before LRC surgery) between the two groups [12]. The incidence of urinary tract complications within 3 years after LRC surgery was also compared between the two groups.

### Statistical analyses

Statistical analyses were performed using JMP Pro 17.2.0 (SAS Institute, Cary, NC, USA). All variables are summarized as medians (ranges) or numbers (percentages). Quantitative variables were compared using the Mann–Whitney U test. Qualitative variables were analyzed using Fisher's exact test or chi-square test, where appropriate. Univariate and multivariate analyses using logistic regression models were performed to predict the incidence of CKD at 3 years after LRC surgery. Since no prior studies have examined the causal relationship between renal function and UD after LRC surgery using uroflowmetry test results, multivariate analyses analyzed the variables with a p-value < 0.05 in the univariate analyses. All reported *p*-values are two-sided, and *p*-values < 0.05 were considered significant.

## Results

### Patient characteristics and urinary tract complications

Table 1 shows the characteristics of the 83 patients included in the final analysis. During hospitalization, seven patients required reinsertion of the urinary catheter, and one began pharmacotherapy. Based on uroflowmetry tests after discharge, there were 21 patients (25%) in the UD group. These patients were not referred to a urologist based on the uroflowmetry test results. The patients in the UD group were significantly older than those in the non-UD group (70 years vs. 61 years; *p* = 0.007). However, no significant differences were observed in the sex, history of diabetes mellitus, BPH, hypertension, and Charlson Comorbidity Index between the two groups [24]. The frequency of LLND (48% vs. 13%; *p* = 0.002) and combined resection of adjacent organs (29% vs. 0%; *p* = 0.0001) was significantly higher in the UD group than in the non-UD group, and operation duration was significantly longer in the UD group than in the non-UD group (417 min vs. 346 min; *p* = 0.03). After LRC surgery, adjuvant therapy was equivalently performed between the two groups.

**Table 1:** Patients’ characteristics and treatment

Variables		non-UD (n = 62)	UD (n = 21)	p-value
Age, year	Median (range)	61 (35–88)	70 (46–81)	0.007
Sex	Male, n (%)	43 (69.4)	12 (57.1)	0.42
	Female, n (%)	19 (30.6)	9 (42.9)	
BMI, kg/m^2^	Median (range)	23.4 (13.9–32.1)	23.2 (17.4–29.7)	0.78
DM	Yes, n (%)	10 (16.1)	3 (14.3)	1.00
Hypertension	Yes, n (%)	11 (17.7)	5 (23.8)	0.53
BPH	Yes, n (%)	4 (6.5)	2 (9.5)	0.64
Charlson comorbidity index	Median (range)	2 (2–6)	2 (2–7)	0.57
Neoadjuvant CRT	Yes, n (%)	35 (56.5)	16 (76.2)	0.13
Type of resection	LAR, n (%)	41 (66.1)	13 (61.9)	0.45
	ISR, n (%)	13 (21.0)	3 (14.3)	
	APR, n (%)	8 (12.9)	5 (23.8)	
Surgical approach	Open, n (%)	1 (1.6)	1 (4.8)	0.65
	Laparoscopic, n (%)	43 (69.4)	13 (61.9)	
	Robot, n (%)	18 (29.0)	7 (33.3)	
Stoma creation	Diverting stoma, n (%)	44 (71.0)	12 (57.1)	0.42
	APR, n (%)	8 (12.9)	5 (23.8)	
	Not created	10 (16.1)	4 (19.0)	
LLND	Yes, n (%)	8 (12.9)	10 (47.6)	0.002
Combined resection of adjacent organs	Yes, n (%)	0 (0.0)	6 (28.6)	0.0001
Pelvic nerve injury	Yes, n (%)	0 (0.0)	2 (9.5)	0.06
Pathological T stage	T0–2, n (%)	42 (67.7)	10 (47.6)	0.12
	T3–4, n (%)	20 (32.3)	11 (52.4)	
Pathological N stage	N0, n (%)	45 (72.6)	14 (66.7)	0.59
	N1–3, n (%)	17 (27.4)	7 (33.3)	
Pathological stage	Stage 0–II, n (%)	44 (71.0)	12 (57.1)	0.29
	Stage III–IV, n (%)	18 (29.0)	9 (42.9)	
Operation time, min	Median (range)	346 (212–692)	417 (232–688)	0.03
Blood loss, mL	Median (range)	100 (0–1850)	150 (1–1130)	0.13
Adjuvant therapy	Yes, n (%)	22 (35.5)	9 (42.9)	0.61

*UD*, Urinary dysfunction; *BMI*, Body mass index; *DM*, Diabetes mellitus; *BPH*, Benign prostate hyperplasia; *CRT*, Chemoradiotherapy; *LAR*, Low anterior resection; *ISR*, Intersphincteric resection; *APR*, Abdominoperineal resection; *LLND*, Lateral lymph node dissection

Table 2 summarizes the incidence of urinary tract complications within 3 years after LRC surgery. Five patients with urinary tract complications were referred to a urologist at our hospital during this period. One patient who complained of frequent urination was diagnosed with urinary retention, another patient was diagnosed with UTI following an examination for fever symptoms, and three patients were diagnosed with hydronephrosis by routine computed tomography. The patient with urinary retention began pharmacotherapy, and two of the three patients with hydronephrosis underwent ureteral stent placement. The incidence of urinary tract complications within 3 years after LRC surgery after discharge was significantly higher in the UD group than in the non-UD group (19% vs. 2%; *p* = 0.01).

**Table 2:** The incidence of urinary tract complications within 3 years after LRC surgery

		non-UD (n = 62)	UD (n = 21)	p-value
Total urinary tract complications	n (%)	1 (1.6)	4 (19.0)	0.01
UTI	n (%)	1 (1.6)	0 (0)	1.00
Urinary retention	n (%)	0 (0)	1 (4.8)	0.25
Hydronephrosis	n (%)	0 (0)	3 (14.3)	0.01

*LRC,* Lower rectal cancer; *UD*, Urinary dysfunction; *UTI*, Urinary tract infection

### Renal function

**Fig. 2:**
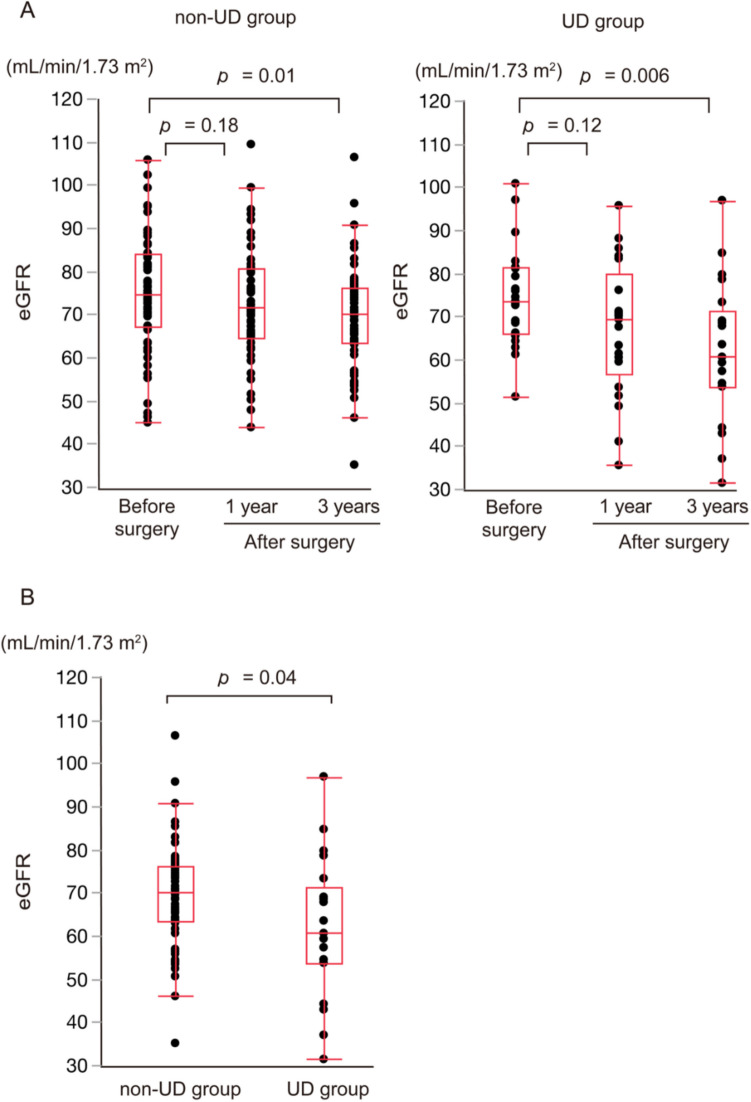
(**A**) Changes in eGFR. (**B**) Comparison of eGFR at 3 years after LRC surgery between the non-UD and UD groups. eGFR: Estimated glomerular filtration rate; UD: Urinary dysfunction; LRC: Lower rectal cancer

**Fig. 3:**
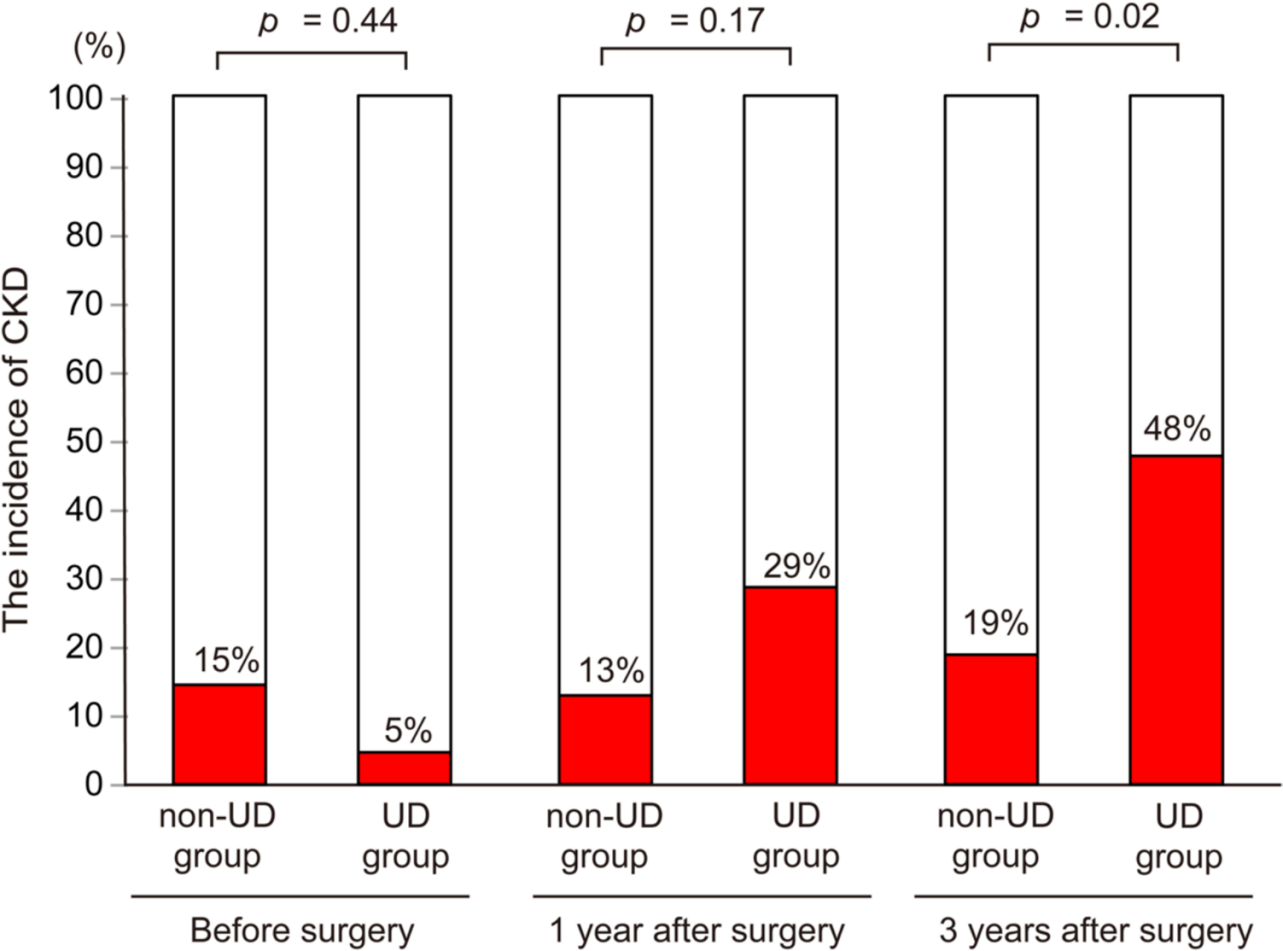
Comparison of the incidence of CKD between the non-UD and UD groups. CKD: Chronic kidney disease; UD: Urinary dysfunction; LRC: Lower rectal cancer

**Fig. 4:**
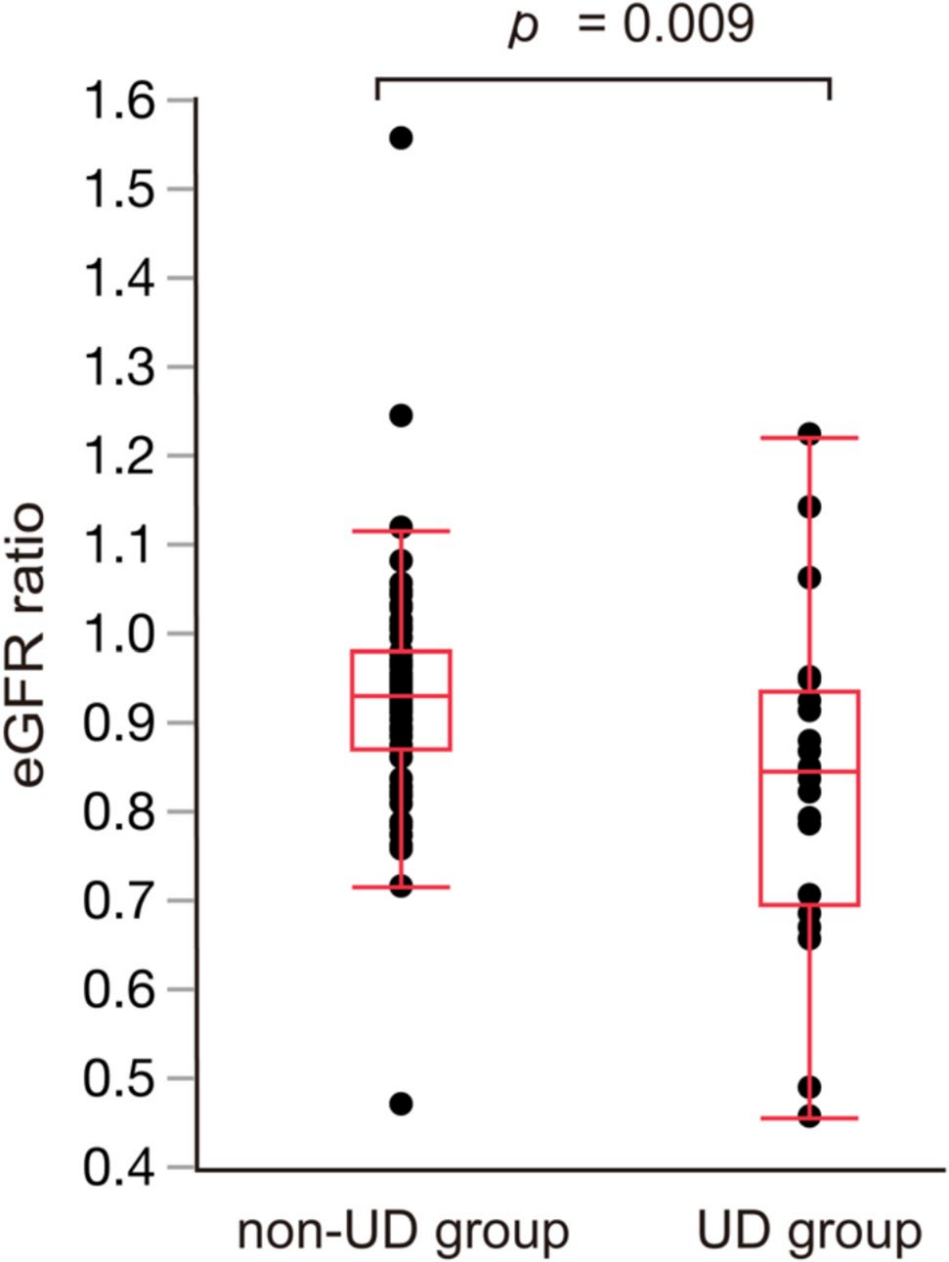
Comparison of eGFR ratio at 3 years after LRC surgery between the non-UD and UD groups eGFR: Estimated glomerular filtration rate; LRC: Lower rectal cancer; UD: Urinary dysfunction

Figure 2A summarizes the changes in eGFR. For both groups, eGFR at 1 year after LRC surgery was lower than preoperative levels and declined further 3 years post-surgery. In comparisons between the groups, eGFR at 3 years after LRC surgery was significantly lower in the UD group than in the non-UD group (60.6 mL/min/1.73 m^2^ vs. 70.1 mL/min/1.73 m^2^; *p* = 0.04; Fig. 2B). Figure 3 shows changes in the incidence of CKD. Consistent with eGFR trends, CKD incidence at 3 years after LRC surgery was significantly higher in the UD group than in the non-UD group (48% vs. 19%; *p* = 0.02). Figure 4 shows the eGFR ratio at 3 years after LRC surgery, which was significantly lower in the UD group than in the non-UD group (0.84 vs. 0.92; *p* = 0.009).

To identify factors associated with CKD at 3 years after LRC surgery, we performed univariate and multivariate analyses, as shown in Table 3 and Online Resource 1. The significant variables identified in univariate analyses (patients’ age (*p* = 0.03), history of hypertension (*p* = 0.001), and UD (*p* = 0.02)) were subjected to multivariate analyses. Both a history of hypertension (odds ratio 5.49, 95% CI 1.62–20.03, *p* = 0.006) and UD (odds ratio 3.27, 95% CI 1.02–10.75, *p* = 0.046) were identified as independent risk factors for CKD at 3 years post-surgery. Additionally, subgroup analyses of the incidence of CKD at 3 years after LRC surgery according to patients’ age (≥ 65 vs. < 65) and history of hypertension (hypertension vs. non-hypertension) are presented in Online Resource 2. Despite patients’ age and history of hypertension, individuals with UD were more likely to progress to CKD at 3 years after LRC surgery than those without UD.

**Table 3:** Univariate and multivariate analysis of CKD at 3 years after LRC surgery

		Univariable		Multivariable	
Variables		*N* (%) of CKD three years after surgery (*n* = 22)	*p*-value	Odds ratio (95% CI)	*p*-value
Patients’ age (years)	≥ 65	15/39 (38.5)	0.03	2.71 (0.89–8.86)	0.08
	< 65	7/44 (15.9)		0.37 (0.11–1.12)	
Sex	Male	13/55 (23.6)	0.44		
	Female	9/28 (32.1)			
DM	Yes	3/13 (23.1)	1.00		
	No	19/70 (27.1)			
Hypertension	Yes	9/16 (56.3)	0.001	5.49 (1.62–20.03)	0.006
	No	13/67 (19.4)		0.18 (0.05–0.62)	
BPH	Yes	2/6 (33.3)	0.65		
	No	20/77 (26.0)			
Neoadjuvant CRT	Performed	12/51 (23.5)	0.46		
	Not performed	10/32 (31.3)			
Type of resection	APR	5/13 (38.5)	0.32		
	Others	17/70 (24.3)			
Stoma creation	Diverting stoma	15/56 (26.8)	1.00		
	Others	7/27 (25.9)			
LLND	Performed	6/18 (33.3)	0.55		
	Not performed	16/65 (24.6)			
Pathological T stage	T3–4	7/31 (22.6)	0.61		
	T0–2	15/52 (28.9)			
Pathological N stage	N1-2	6/24 (25.0)	1.00		
	N0	16/59 (27.1)			
Operation time (min)	≥ 350	14/43 (32.6)	0.22		
	< 350	8/40 (20.0)			
Blood loss (mL)	≥ 100	13/49 (26.5)	1.00		
	< 100	9/34 (26.5)			
Adjuvant therapy	Performed	8/31 (25.8)	1.00		
	Not performed	14/52 (26.9)			
UD	Yes	10/21 (47.6)	0.02	3.27 (1.02–10.75)	0.046
	No	12/62 (19.4)		0.31 (0.09–0.98)	

*CKD*, Chronic kidney disease; *LRC*, Lower rectal cancer; *DM*, Diabetes mellitus; *BPH*, Benign prostate hyperplasia; *CRT*, Chemoradiotherapy; *APR*, Abdominoperineal resection; *LLND*, Lateral lymph node dissection; *UD*, Urinary dysfunction

## Discussion

We retrospectively investigated the influence of UD after LRC surgery on renal function in patients treated at our hospital. Of the 83 patients, 21 (25%) were classified into the UD group. Regarding renal function, eGFR at 3 years after LRC surgery was significantly lower in the UD group than in the non-UD group. Additionally, the incidence of urinary tract complications within 3 years after LRC surgery was significantly higher in the UD group than in the non-UD group. Furthermore, UD was identified as an independent risk factor for CKD at 3 years after LRC surgery.

Owing to the influence of patient age, the eGFR in both groups significantly decreased at 3 years after LRC surgery. However, a significant number of patients with UD showed a decrease in eGFR compared to those without UD. Although the causal relationship between renal dysfunction and UD after LRC surgery remains unclear [12, 13], our analyses using uroflowmetry test results showed that UD after LRC surgery might adversely affect renal function similar to the impact observed in BPH [8, 9]. In addition, UD occurred significantly more frequently in older patients and those who underwent LLND, combined resection of adjacent organs, or prolonged operations. These findings underscore the importance of monitoring urinary function in these patient populations.

In the current study, uroflowmetry tests were performed within 3 months, and we used PVR values to identify patients with UD [20]. Our findings revealed that UD was significantly associated with a decrease in eGFR and progression to CKD at 3 years after LRC surgery. Therefore, postoperative uroflowmetry tests may be helpful in evaluating urinary function after LRC surgery and predicting the risk of renal dysfunction. However, it remains unclear whether the high PVR observed within 3 months after LRC surgery persists over time. There have been several reports of long-term UD in pelvic cancer, such as rectal and uterine cancer [4, 7, 11, 25]; however, further research is needed to determine whether high PVR persists after LRC surgery. Additionally, post-discharge PVR measurement is not a routine procedure in most institutions, whereas PVR measurement is routinely performed during hospitalization after LRC surgery. In this study, 20 of the 21 patients diagnosed with UD were identified through uroflowmetry tests performed after discharge. Therefore, evaluation of urinary function both during hospitalization and post-discharge may be recommended. Further research is also needed to examine whether urogenital treatments, such as medication, self-catheterization, and reinsertion of a urinary catheter [26], can improve the renal function of post-LRC surgery patients diagnosed with UD after discharge.

For patients with LRC, performing LRC surgery that minimizes the risk of UD may help prevent postoperative renal dysfunction. Although UD remains a major complication after LRC surgery, previous studies have reported that robotic surgery is superior for preventing UD during hospitalization [3]. Further studies are needed to investigate the usefulness of LRC robotic surgery for preventing persistent UD.

The present study has some limitations. First, our analyses had substantial limitations due to the retrospective, single-center design and a small sample size. Second, patients who did not undergo uroflowmetry after discharge (n = 57) or were lost to follow-up (n = 17) were excluded. Third, uroflowmetry tests were performed only once within 3 months after LRC surgery. To assess whether patients with UD continue to have a high PVR, periodic evaluations, such as annual testing, should be conducted throughout the observation period. Finally, uroflowmetry is more reliable when the voided volume exceeds 150 mL in urology [14, 19]; however, owing to stoma creation [21], several patients could not meet this threshold.

Despite these limitations, to our knowledge, this is the first study to report that UD can negatively impact renal function after LRC surgery. This study’s novelty lies in demonstrating the relationship between renal function and UD after LRC surgery using the results obtained from uroflowmetry tests, which are considered a reliable evaluation method of urinary function [19].

In conclusion, patients with UD comprised 25% of all patients in this study. Their renal function significantly worsened, and they were more likely to progress to CKD at 3 years after LRC surgery. Our findings demonstrated that UD after LRC surgery was likely to cause renal dysfunction, emphasizing the need for evaluation and management both during hospitalization and after discharge.

## Supplementary Information

Below is the link to the electronic supplementary material.

Supplementary file1 (PDF 230 KB)

Supplementary file2 (PDF 327 KB)

## Data Availability

No datasets were generated or analysed during the current study.
