# Supplementary material for: Association of urinary dysfunction after lower rectal cancer surgery with renal function: a single-center study
Source: Int J Colorectal Dis. 2025 Jul 14;40(1):158. doi: 10.1007/s00384-025-04955-1 (PMC12259756; doi:10.1007/s00384-025-04955-1)
Supplement: Supplementary file 1 — Supplementary file1 (PDF 230 KB) [file 384_2025_4955_MOESM1_ESM.pdf]

Association of urinary dysfunction after lower rectal cancer surgery with renal function: A single-center study

International Journal of Colorectal Disease

Ryosuke Kikuchi[1], Kazuhito Sasaki[1*], Yusuke Sato[2], Aya Niimi[2], Akira Sakamoto[1], Hiroaki Nozawa[1], Koji Murono[1], Shigenobu Emoto[1], Yuichiro Yokoyama[1], Kensuke Kaneko[1], Haruki Kume[2], and Soichiro Ishihara[1]

[1]Department of Surgical Oncology, Faculty of Medicine, The University of Tokyo, Tokyo, Japan

[2]Department of Urology, Faculty of Medicine, The University of Tokyo, Tokyo, Japan

*Corresponding author: Kazuhito Sasaki, E-mail address: sasakik-tky@umin.ac.jp

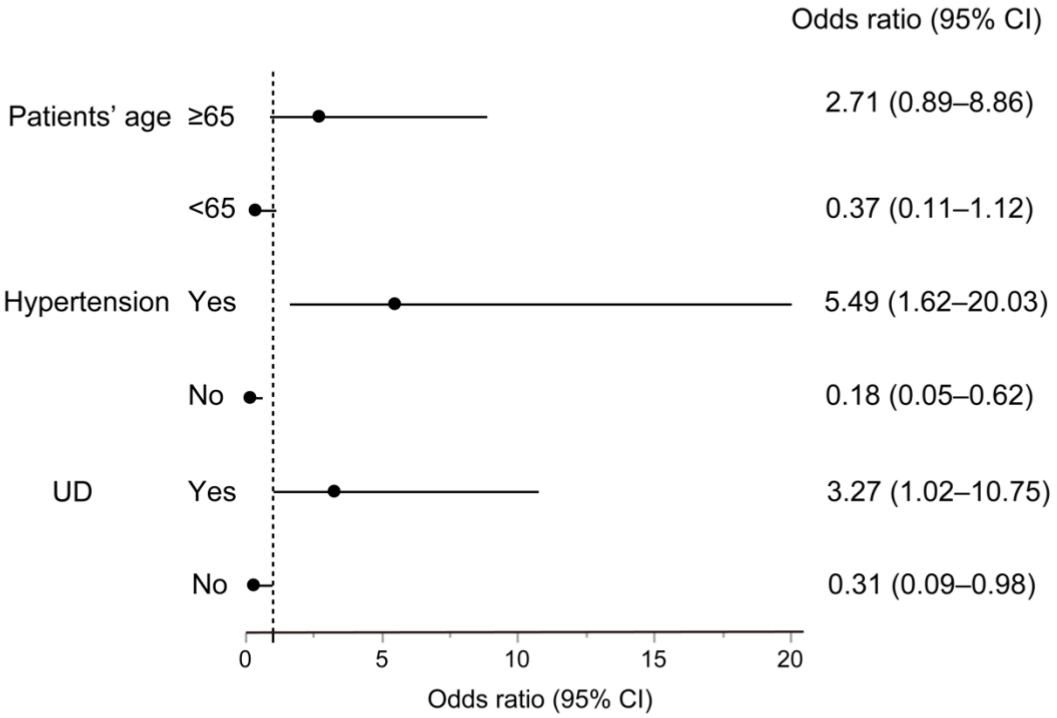

Online Resource 1. Forest plot—multivariate analyses for CKD at 3 years after LRC surgery. CKD: Chronic kidney disease; LRC: Lower rectal cancer; UD: Urinary dysfunction.
