# Supplementary material for: Association of urinary dysfunction after lower rectal cancer surgery with renal function: a single-center study
Source: Int J Colorectal Dis. 2025 Jul 14;40(1):158. doi: 10.1007/s00384-025-04955-1 (PMC12259756; doi:10.1007/s00384-025-04955-1)
Supplement: Supplementary file 2 — Supplementary file2 (PDF 327 KB) [file 384_2025_4955_MOESM2_ESM.pdf]

Association of urinary dysfunction after lower rectal cancer surgery with renal function: A single-center study

International Journal of Colorectal Disease

Ryosuke Kikuchi[1], Kazuhito Sasaki[1*], Yusuke Sato[2], Aya Niimi[2], Akira Sakamoto[1], Hiroaki Nozawa[1], Koji Murono[1], Shigenobu Emoto[1], Yuichiro Yokoyama[1], Kensuke Kaneko[1], Haruki Kume[2], and Soichiro Ishihara[1]

[1]Department of Surgical Oncology, Faculty of Medicine, The University of Tokyo, Tokyo, Japan

[2]Department of Urology, Faculty of Medicine, The University of Tokyo, Tokyo, Japan

*Corresponding author: Kazuhito Sasaki, E-mail address: sasakik-tky@umin.ac.jp

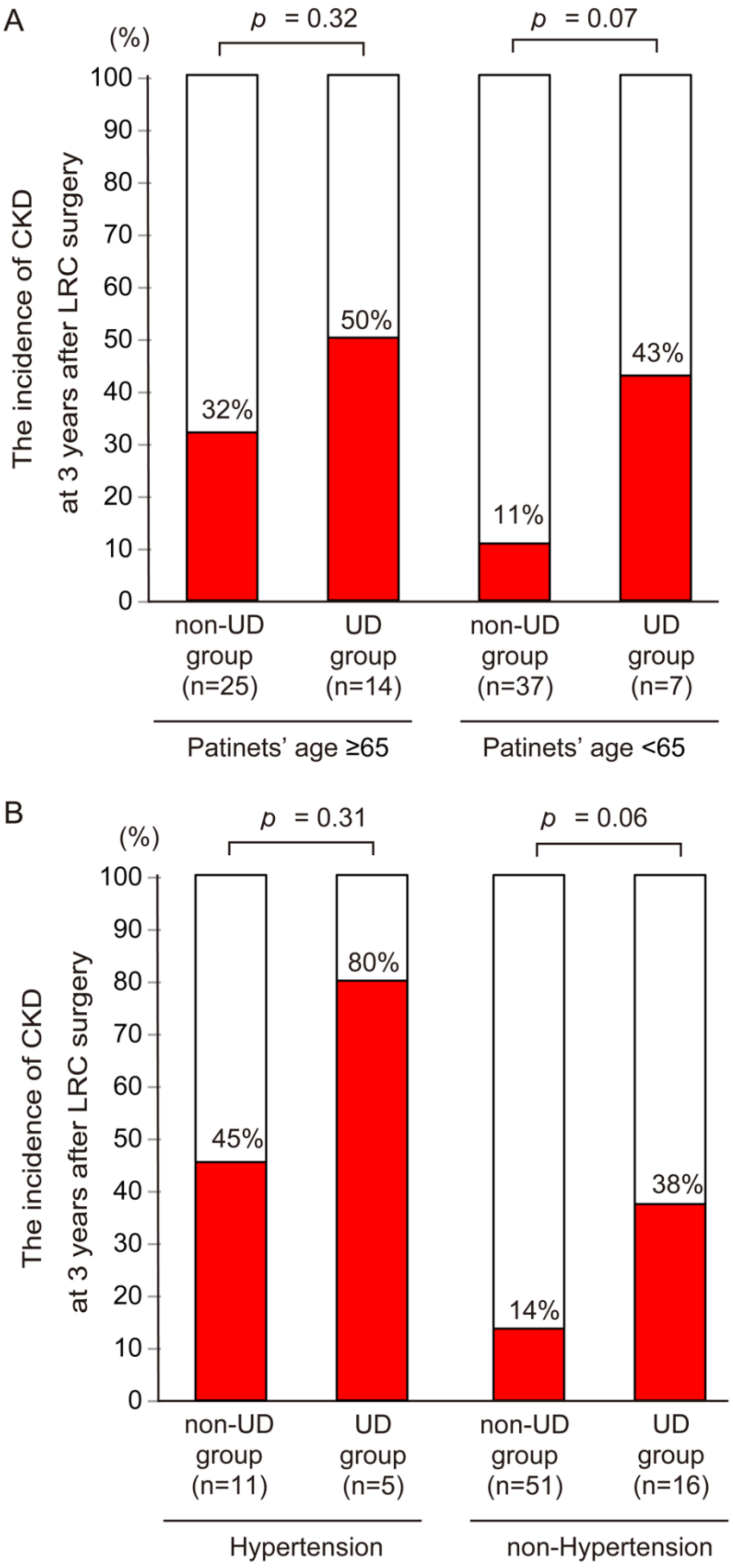
A
(%)
p = 0.32
p = 0.07
The incidence of CKD at 3 years after LRC surgery
100
90
80
70
60
50
40
30
20
10
0
32%
50%
11%
43%
non-UD group (n=25)
UD group (n=14)
non-UD group (n=37)
UD group (n=7)
Patinets' age ≥65
Patinets' age <65
B
(%)
p = 0.31
p = 0.06
The incidence of CKD at 3 years after LRC surgery
100
90
80
70
60
50
40
30
20
10
0
45%
80%
14%
38%
non-UD group (n=11)
UD group (n=5)
non-UD group (n=51)
UD group (n=16)
Hypertension
non-Hypertension

Online Resource 2. Subgroup analyses of the incidence of CKD at 3 years after LRC surgery according to (A) patients' age (≥65 vs. <65) and (B) history of hypertension (hypertension vs. non-hypertension). CKD: Chronic kidney disease; LRC: Lower rectal cancer; UD: Urinary dysfunction.
